# Supplementary material for: Increasing wintertime cloud opacity increases surface longwave radiation at a long-term Arctic observatory
Source: Nat Commun. 2025 Nov 1;16:9135. doi: 10.1038/s41467-025-64441-8 (PMC12579616; doi:10.1038/s41467-025-64441-8)
Supplement: Supplementary file 1 — Supplementary Information [file 41467_2025_64441_MOESM1_ESM.pdf]

Supporting information for "Increasing wintertime cloud opacity increases surface longwave radiation at a long-term Arctic observatory"

Leah Bertrand[1,2], Jennifer E. Kay[1,2], and Gijs de Boer[3]

[1] Department of Atmospheric and Oceanic Sciences, University of Colorado, Boulder, 311 UCB, Boulder, 80309, CO, USA

[2] Cooperative Institute for Research in Environmental Sciences, University of Colorado, Boulder, 216 UCB, Boulder, 80309, CO, USA.

[3] Environmental and Climate Sciences Department, Brookhaven National Laboratory, 98 Rochester St, Upton, 11973, NY, USA.

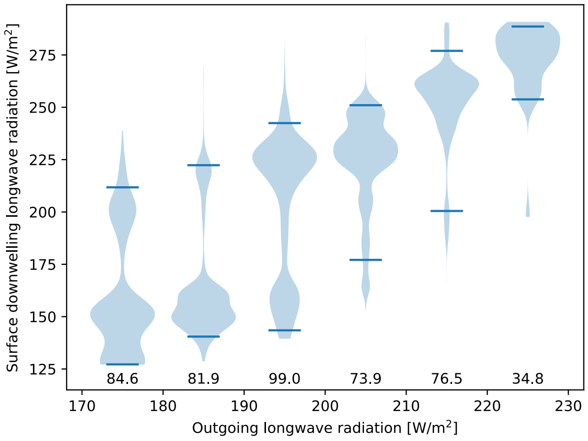

Supplementary Figure 1. Wintertime ARM NSA distributions of surface downwelling longwave binned by top-of-atmosphere outgoing longwave radiation (OLR). Bins are spaced at 10 W/m2, and lines indicate 5th and 95th percentiles. Text indicates difference between 95th and 5th percentiles for each OLR bin.

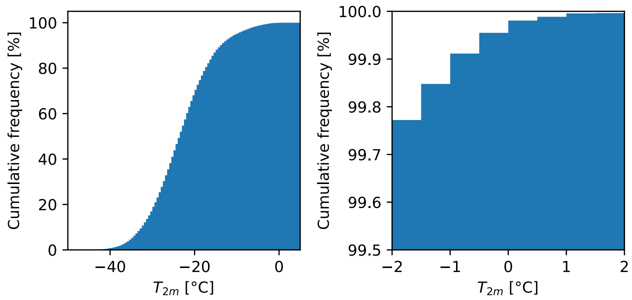

Supplementary Figure 2. Minute-frequency cumulative distribution function of December-March near-surface air temperature at ARM NSA, 1998-2023.

Supplementary Note 1

In our analysis, we assume that the Planck feedback is the only process driving the surface upward longwave response to warming. Here, we justify that assumption. The upward surface longwave response to warming is

$$\frac{dF_U}{dT_a} = 4\,\sigma T_s^3\,\frac{dT_s}{dT_a}, \qquad \text{(S1)}$$

where F_U is surface upwelling longwave radiation, T_a is near-surface air temperature, and T_s is surface skin temperature. We assume a constant surface emissivity of 1.

If other processes (changes in sensible heat, latent heat, subsurface heat flux) were to influence the upward longwave response to warming, it would occur via dT_s/dT_a. If these other processes suppress dF_U/dT_a (e.g., via melting), then $dT_s/dT_a < 1$. If these other processes accelerate dF_U/dT_a (e.g., via increasing downward sensible heat), then $dT_s/dT_a > 1$. Assuming that the Planck feedback is the only driver of the upward longwave response to warming means assuming $dT_s/dT_a = 1$. In other words,

$$\left.\frac{dF_U}{dT_a}\right|_{planck} = 4\,\sigma T_s^3\,. \qquad \text{(S2)}$$

To test our assumption, we compare equation (2) to the observed dF_U/dT_a of 3.61 $\pm$ 0.17 W/m^2/K (Main text figure 1b). To evaluate equation (2), we calculate T_s from observed monthly mean F_U according to $T_s = \left(\frac{F_U}{\sigma}\right)^{1/4}$. Then we can make a prediction for $dF_U/dT_a|_{planck}$ based on each monthly-mean surface skin temperature (Figure S3). The uncertainty in our expected overall value for $dF_U/dT_a|_{planck}$ is then given by the range of individual estimates. Since $dF_U/dT_a|_{planck}$ (Figure S3, black lines) is consistent with the observed dF_U/dT_a (Figure S3, red lines), we conclude that our Planck-only hypothesis explains the surface upward longwave response to warming.

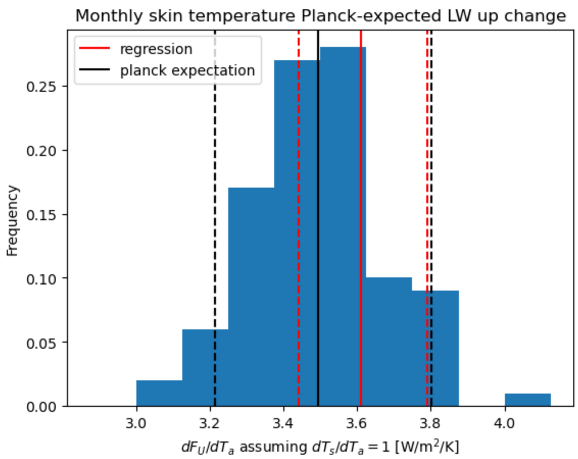

Supplementary Figure 3. Planck feedback expectation for surface upward longwave radiation response to warming, with average value and 95% confidence intervals for observed (red) and predicted (black) change. Blue bars show histogram of expected values calculated from equation (S2) from each month of observations.

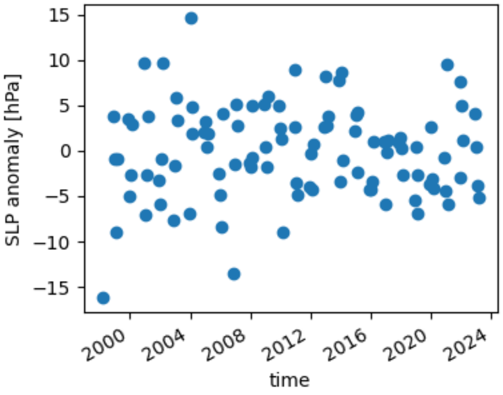

Supplementary Figure 4. Monthly wintertime (DJFM) local sea level pressure anomalies over time. Linear regression has a p-value of 0.86 and an r-value of 0.02 (no trend).

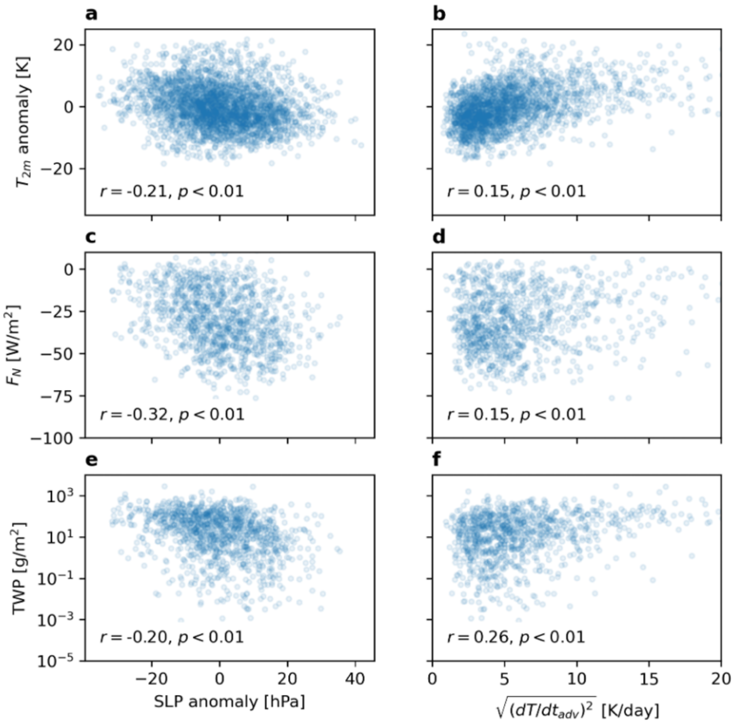

Supplementary Figure 5. Daily relationships (ordinary least-squares regression) between confounder variables (sea level pressure, advective warming) and quantities used in main text Figure 4. These quantities are near-surface air temperature anomaly (a,b), net surface longwave flux (c,d), and total water path (e,f). Confounder variables are local sea level pressure anomaly (a,c,e) and advective warming from reanalysis (b,d,f). Advective warming is defined as the daily standard deviation in advective temperature tendency from ERA5 renalysis. The standard deviation (rather than the average) is used because average advective temperature tendency has no correlation with daily average surface temperature.

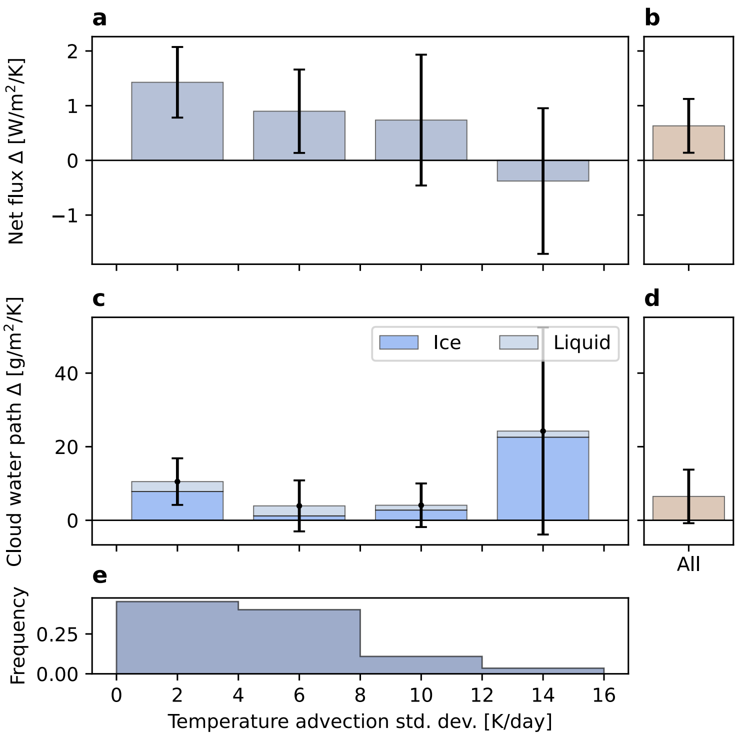

Supplementary Figure 6. Like manuscript Figure 4 but using daily standard deviation of advective temperature tendency from ERA5 reanalysis instead of local surface pressure. (a-b) Net surface flux and (c-d) cloud water path response to warming with (a,c) and without (b,d) controlling for circulation variability. (e) Frequency of each local advective temperature tendency bin. Bars show 95% confidence interval for regression slopes. (c) Total and individual ice and liquid contributions to cloud water path.

Statistic	2004	2005	2006	2008	2009	2010	2011	2014	2015	2016	2017	2018	2019
**Median**	-3.91	-6.06	-5.79	-1.95	-3.50	-2.09	-1.79	-2.89	-2.03	-3.55	-1.27	0.09	1.00
**IQR**	10.06	14.92	18.80	9.38	10.66	11.51	13.86	9.18	14.92	10.42	17.69	12.07	16.18

Supplementary Table 1. Study closure statistics (calculated minus observed) for surface downwelling longwave radiation by year. All values in W/m^2.

Citation	Study duration	Median (W/m^2)	IQR (W/m^2)
Present study	13 years	-2.6	13.1
Shupe et al. 2015	2 years	2.3	10.1
Ebell et al. 2020	3 years	0.7	12.2
Griesche et al. 2024	1 month	-3	8
Barrientos-Velasco et al. 2023	3 days	-19	Unknown

Barrientos-Velasco et al. 2024	1 year	3.2	18.8*

Supplementary Table 2. Literature review of closure statistics for surface downwelling longwave radiative flux from Arctic surface-based observations.

* Converted from standard deviation assuming normal distribution (IQR = 1.34σ)
